# Extraction of a Central Incisor From the Plica Fimbriata in the Outpatient Family Medicine Office: What’s That Rattle?

**DOI:** 10.7759/cureus.36714

**Published:** 2023-03-26

**Authors:** Shawn Y Sunu, Jason R Woloski

**Affiliations:** 1 Family Medicine Residency Program, Geisinger Health System, Wilkes-Barre, USA; 2 Family Medicine Residency Program, Geisinger Health System, Geisinger Commonwealth School of Medicine, Wilkes-Barre, USA

**Keywords:** all-terrain vehicle (atv), extraction, tooth, plica fimbriata, central incisor

## Abstract

All-terrain vehicle (ATV) accidents are all too common in the United States and can result in long-term injuries. Therefore, proper after-care is essential for the recovery of an injured person. Here, we present a case where an embedded tooth was overlooked for almost an entire year after an ATV accident. No imaging was performed despite multiple clinic and emergency department visits. The tooth was not discovered to be embedded within the tongue until it later started migrating and protruding. Therefore, extraction was performed in the office.

## Introduction

Based on a 2021 U.S. Consumer Product Safety Commission annual report, an estimated 526,900 emergency department-treated injuries were associated with off-highway vehicles between 2016 and 2020. These vehicles include all-terrain vehicles (ATVs), recreational off-highway vehicles (ROV), and utility-terrain vehicles (UTVs). Of these injuries, 96% are reportedly related to ATVs [1].

Studies have also demonstrated that irrespective of the mechanism of injury, traumatic dental injuries are prevalent in the U.S., with estimates of one in four Americans experiencing a traumatic dental injury (TDI) [2-3]. Similarly, a retrospective study examining athletes discovered that the most common type of orofacial injury sustained was in relation to soft-tissue injuries, such as lacerations [4-5]. The same study also showed that the most common sites of injuries included the lip, maxilla, and central maxillary incisors [4-6]. Therefore, a thorough evaluation of the lips, oral mucosa, gingiva, and frenula for lacerations, hematomas, and embedded tooth fragments should be done promptly by a medical professional after an ATV injury to ensure proper healing of the soft tissues and identify any tooth involvement. This is further outlined in the guidelines for managing traumatic dental injuries by the International Association of Dental Traumatology [7].

## Case presentation

A 28-year-old female with no significant past medical history presented to an outpatient family medicine clinic with the chief complaint of "rattle." When asked, she believed that her symptoms most likely stemmed from an ATV accident ten months prior. During her accident, she believed a tooth fragment traumatically broke off and may have been embedded in her tongue. However, when she went to the emergency department, a physical exam only revealed a fragmented lower central incisor, a bruised lip with a 0.5-centimeter (cm) laceration, and a puncture wound on the left side of her tongue. There was no mention of an embedded tooth at the time or further exploration to identify the tooth fragment. As the patient was medically stable without any airway or breathing concerns, she was discharged with clindamycin and advised to follow up with a dentist.

At her dental visit a couple of weeks later, the missing fragment of her lower central incisor was not able to be located and was suspected to have been aspirated at the time of her accident. The dentist replaced the missing fragment, and no further recommendations were given.

The patient had several contacts with the healthcare system in the months to follow. However, most visits focused on the continued recommendation to follow up with dental medicine (despite the patient’s repeated reports of scheduling difficulties) and the initiation of antibiotics and anti-inflammatories for source control.

Ten months after her ATV accident, the patient reported biting down on her tongue and feeling a "lumpy sensation." Although initially dismissed by the patient as inconsequential, the patient later reported the sensation transitioning to more of a "rattle" sound, which was also noticed by family members. The sound occurred when she moved her tongue laterally over her neighboring teeth.

Over the next few days, the patient soon felt a "hard sensation" in a portion of her tongue. She began to suspect that a fragment of her original tooth was embedded in her tongue from her prior ATV accident. The patient was ultimately able to visualize a white, calcified entity under the tongue. She reported an unsuccessful attempt at home to extract the suspected tooth fragment at home using forceps. Hence, she scheduled a visit to the family medicine clinic for further discussion and potential removal. At the time of the appointment, the patient reported to the physician that her general dentist did not treat embedded teeth when calling for an appointment.

**Figure 1:**
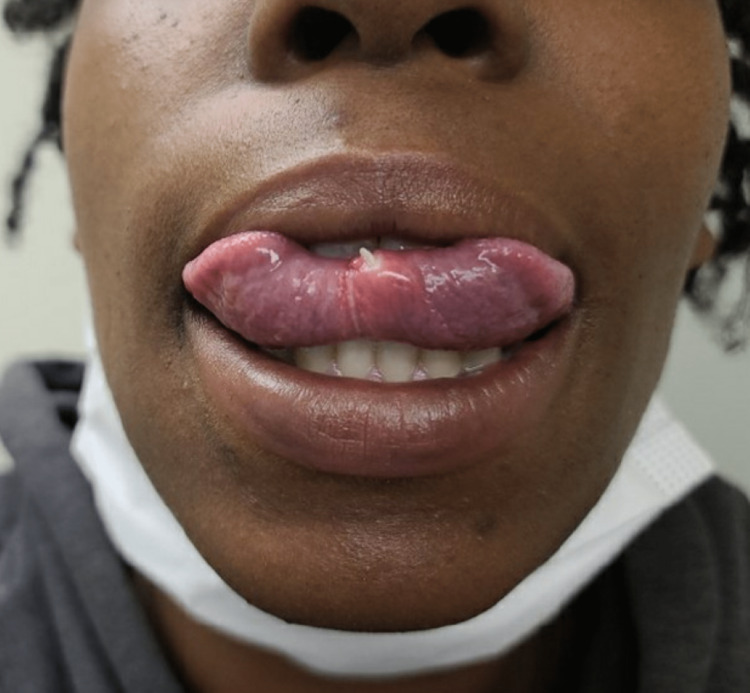
Small white protrusion lateral to the lingual frenulum

**Figure 2:**
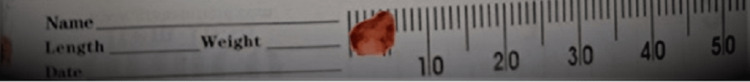
A 5-millimeter (mm) fragmented tooth

**Figure 3:**
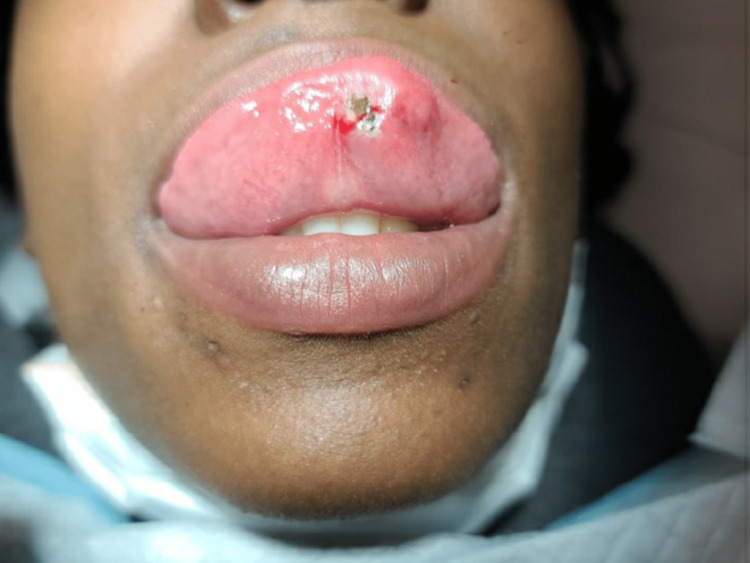
Post-extraction cauterization

During her outpatient family medicine visit, the patient denied having any fevers, nausea, vomiting, or pain. On physical exam, her vital signs were stable, and a small, firm white protrusion was noted lateral to the lingual frenulum (Figure 1). Given the object’s appearance, the differential included a fragmented tooth, a calcified mass, a possible migrated tonsil stone embedded into a secondary site, or an otherwise unidentified foreign object. However, given the patient’s history and physical exam, the most likely diagnosis was determined to be the previously missing avulsed tooth fragment. A consent form for the extraction of the fragmented tooth was then discussed and signed. As such, initial attempts to extract the fragment were performed using forceps. However, the fragmented portion was deeper than initially suspected. Therefore, 1 ml of 1% lidocaine was injected under the tongue near the embedded tooth in preparation for a small incision. Afterward, a 10-blade scalpel was used to break the adhesions near the tooth, and a hemostat was eventually used to extract a 5-mm fragmented tooth (Figure 2). Gauze and a quick dab of silver nitrate were used to stop the bleeding after the extraction (Figure 3). On discharge from the clinic, instructions were given for the patient to use saltwater gargle rinses and to watch for fevers, chills, or swelling. The patient ultimately tolerated the procedure well without complication.

## Discussion

From an American Academy of Pediatric Dentistry (AAPD) recommendation in 2010 and a Turkish study in 2002, it was noted that the use of protective gear, such as mouthguards, could redistribute the force of impact in sports and reduce the risk of severe orofacial injury [8-10]. Although, in this case, the patient drove an ATV as a recreational activity, the use of a mouthguard may have been warranted given the after-effects of her accident. Therefore, whether it be recreational off-road travel or a competitive extreme sports athlete, proper protective equipment such as mouthguards should be advised as preventative measures at yearly visits.

In the event that a dental trauma injury does occur, prompt removal of an embedded fragment is important to prevent long-term effects such as cosmetic defects or recurrent infections. Therefore, it is important to perform a thorough investigation of the oral cavity after trauma, especially when dental trauma has occurred. If a tooth fragment cannot be located after a thorough history and physical examination, then imaging should be considered, whether it be a panoramic dental X-ray or dental cone beam computed tomography.

Once an embedded tooth has been located, initial management should ensure the patient is stable and determine whether antibiotics are required. Given the high degree of bacteria located in the oral cavity, if the patient has any systemic symptoms, antibiotics should be considered. It should then be determined if the tooth can be extracted safely in an outpatient setting. If not, an appropriate referral to an oral and maxillofacial surgeon should be made.

## Conclusions

Given the known prevalence of traumatic dental injuries, providers should perform a thorough examination of the lips, oral mucosa, gingiva, and frenula. As noted in this case report, any missing or chipped dentition from an injury should prompt further evaluation for subsequent migration of the tooth or a tooth fragment to a secondary site, such as the tongue.
